# Gene expression profiling in hepatic tissue of newly weaned pigs fed pharmacological zinc and phytase supplemented diets

**DOI:** 10.1186/1471-2164-9-421

**Published:** 2008-09-17

**Authors:** Michelle M Martínez-Montemayor, Gretchen M Hill, Nancy E Raney, Valencia D Rilington, Robert J Tempelman, Jane E Link, Christopher P Wilkinson, Antonio M Ramos, Catherine W Ernst

**Affiliations:** 1 Department of Anatomy and Cell Biology, Universidad Central del Caribe, Bayamón, PR; 2 McDonald Observatory, College of Natural Sciences, The University of Texas at Austin, Austin, TX, USA; 3 Animal Breeding and Genomics Centre, Wageningen University, Wageningen, The Netherlands; 4 Department of Animal Science, Michigan State University, East Lansing, MI, USA

## Abstract

**Background:**

Zinc (Zn) is an essential trace element. However, Zn bioavailability from commonly consumed plants may be reduced due to phytic acid. Zn supplementation has been used to treat diarrheal disease in children, and in the U.S. swine industry at pharmacological levels to promote growth and fecal consistency, but underlying mechanisms explaining these beneficial effects remain unknown. Moreover, adding supplemental phytase improves Zn bioavailability. Thus, we hypothesized that benefits of pharmacological Zn supplementation result from changes in gene expression that could be further affected by supplemental phytase. The goal of this study was to investigate the effects of feeding newly weaned pigs dietary Zn (150, 1,000, or 2,000 mg Zn/kg) as Zn oxide with or without phytase [500 phytase units (FTU)/kg] for 14 d on hepatic gene expression. Liver RNA from pigs fed 150, 1,000, or 2,000 mg Zn/kg, or 1,000 mg Zn/kg with phytase (n = 4 per treatment) was reverse transcribed and examined using the differential display reverse transcription polymerase chain reaction technique. Liver RNA from pigs fed 150 or 2,000 mg Zn/kg (n = 4 per treatment) was also evaluated using a 70-mer oligonucleotide microarray.

**Results:**

Expressed sequence tags for 61 putatively differentially expressed transcripts were cloned and sequenced. In addition, interrogation of a 13,297 element oligonucleotide microarray revealed 650 annotated transcripts (FDR ≤ 0.05) affected by pharmacological Zn supplementation. Seven transcripts exhibiting differential expression in pigs fed pharmacological Zn with sequence similarities to genes encoding *GLO1*, *PRDX4*, *ACY1*, *ORM1*, *CPB2*, *GSTM4*, and *HSP70.2 *were selected for confirmation. Relative hepatic *GLO1 *(*P *< 0.0007), *PRDX4 *(*P *< 0.009) and *ACY1 *(*P *< 0.01) mRNA abundances were confirmed to be greater in pigs fed 1,000 (n = 8) and 2,000 (n = 8) mg Zn/kg than in pigs fed 150 (n = 7) mg Zn/kg. Relative hepatic *HSP70.2 *(P < 0.002) mRNA abundance was confirmed to be lower in pigs fed 2,000 mg Zn/kg than in pigs fed 150 or 1,000 mg Zn/kg.

**Conclusion:**

Results suggest that feeding pharmacological Zn (1,000 or 2,000 mg Zn/kg) affects genes involved in reducing oxidative stress and in amino acid metabolism, which are essential for cell detoxification and proper cell function.

## Background

Differential gene expression is responsible for morphological and phenotypical differences among animals, since the transcriptome is dynamic [1]. Studies on how micronutrients affect gene expression will help to clarify the role of trace elements in health and metabolism and their connection to biochemical events [2]. The trace mineral zinc (Zn) is involved in gene expression in numerous ways including DNA replication, RNA transcription, through the activity of transcription factors, DNA and RNA polymerases, signal transduction, oxidative stress and playing a role in programmed cell death [3]. It was recently estimated that almost 3,000 different proteins encoded by the human genome bind zinc [4]. An example of such proteins is metallothionein (MT), which exerts a protective effect against stress by acting as an antioxidant, and as a Zn storage and transfer protein [5]. Furthermore, the transcriptional regulation of the MT gene by dietary Zn has been demonstrated in rats [6].

In developing countries Zn deficiency is caused by ingestion of high cereal protein diets rich in phytic acid, an organic form of phosphorus (P) [7]. In addition, common animal feedstuffs contain 60–80% of the total P in the phytic acid molecule, which make Zn unavailable for absorption [8]. Rats fed diets containing 3,000 mg Zn/kg and phytic acid absorb less and excrete more Zn [9]. This may be explained by the formation of mineral-phytic acid complexes, plus the negligible amount of endogenous phytase production in mammals. Therefore, the tendency to excrete phytic acid bound minerals in the feces is increased, and mineral absorption is decreased. Phytase supplementation studies in pigs demonstrate increased plasma Zn concentration [10,11], greater Zn retention [12], and improved bone mineralization [13], while adding phytase to human diets also results in increased iron absorption [14]. Thus, phytase supplementation provides a potential solution to improve Zn availability.

In humans, Zn supplementation has been shown to reduce the morbidity of diarrhea [15], respiratory disease [16], and nematode infection [17]. Moreover, high Zn supplementation to weaned pigs has been successful in increasing growth and decreasing diarrhea [18], while feeding pharmacological Zn (3,000 mg Zn/kg) to weaned pigs improves fecal consistency and also intestinal morphology by increasing villous height and reducing crypt depths of the duodenum and jejunum [19]. However, molecular analyses of the effects of pharmacological Zn to explain the favorable symptoms observed when Zn is supplemented in high quantities have not been undertaken.

We previously reported that feeding the recommended amounts of phytase (500 FTU/kg) and pharmacological Zn (1,000 – 2,000 mg/kg) to newly weaned pigs increased *MT *mRNA abundance and protein concentrations in the liver, kidney and intestinal mucosa [11]. Thus, additional genes are also likely to be affected by this dietary intervention and serve as the basis for the improved health effects obtained with pharmacological Zn supplementation. Therefore, the objective of this experiment was to determine the identity of genes that are differentially expressed in the liver of pigs fed diets with or without pharmacological Zn, and to determine if phytase supplementation further affected gene expression by using differential display reverse transcription polymerase chain reaction (DDRT-PCR) and oligonucleotide microarray technologies. In this paper we report 61 amplicons identified by DDRT-PCR and 650 annotated genes (FDR ≤ 0.05) identified by microarray analysis with putative differential gene expression, of which 5 transcripts with roles in oxidative stress reduction and amino acid metabolism were confirmed to have differential expression in pigs fed pharmacological Zn diets.

## Results

### Identification of expressed sequences displayed by DDRT-PCR

A total of 66 putatively differentially expressed amplicons were cloned and sequenced. High quality nucleotide sequence data was obtained for 61 of these expressed sequence tags (ESTs) and all were submitted to the GenBank database [GenBank:CB826605 – CB826610, GenBank:CF106636 – CF106687, GenBank:DW177047 – DW177050]. A search of the EMBL/GenBank and TIGR databases revealed that 29 of the ESTs (48%) had significant similarities to genes with known identities, 7 were similar to mitochondrial DNA (11%), and the 25 remaining sequences (41%) had unknown identities (Table 1). Identified genes were similar to genes involved in signal transduction (48%), nucleic acid metabolism (17%), oxidative stress response (11%), protein and amino acid metabolism (10%), blood coagulation (7%), and structural roles (7%). Various mRNA abundance patterns were observed among the dietary treatment groups (Table 1) and 5 genes were selected for subsequent confirmation analyses.

**Table 1:** List of differentially expressed products and sequence identity information from differential display reverse transcription PCR.

**EST ID (Length – bp)**	**Sequence ID^a^**	**Species/Accession No.^b^**	**Porcine TC number^c^**	**Pattern of transcript abundance^d^**
***Signal Transduction Genes***				

CF106654 (152)	Activator heat shock 90 kDa protein ATPase homologue 1 (*AHSA1*)	B. taurus/NM_001034666.1	TC243227	3 = 4 > 2 > 1
CF106663 (151)	Activator heat shock 90 kDa protein ATPase homologue 1 (*AHSA1*)	B. taurus/NM_001034666.1	TC243227	3 = 4 > 2 > 1
CF106649 (240)	Golgi autoantigen, golgin subfamily b, macrogolgin (with transmembrane signal) 1, (*GOLGB1*)	H. sapiens/NM_004487.3	TC269106	1 > 2 = 3 = 4
CF106669 (441)	Interferon induced with helicase C domain 1 (*IFIH1*)	H. sapiens/NM_022168.2	TC281891	3 > 4 > 2 > 1
CB826605 (152)^e^	N-myc downstream regulated gene 1 (*NDRG1*)	B. taurus/NM_001035009.1	TC257462	1 > 4
CB826606 (130)^e^	Orosomucoid 1 (*ORM1*)	S. scrofa/PIGA1AG	TC273225	1 > 4
CF106636 (484)	Orosomucoid 1 (*ORM1*)	S. scrofa/PIGA1AG	TC273225	4 > 3 > 2 = 1
CF106686 (546)	Orosomucoid 1 (*ORM1*)	S. scrofa/PIGA1AG	TC273225	3 = 4 > 2 = 1
CF106640 (240)	Progesterone receptor membrane component 1 (*PGRMC1*)	S. scrofa/NM_213911.1	TC238451	4 = 3 > 2 > 1
CF106680 (272)	Progestin and adipoQ receptor family member VII (*PAQR7*)	S. scrofa/NM_213739.1	TC238374	3 > 4 > 2 = 1
CF106679 (354)	Ras, member oncogene family (*RAB2A*)	C. familiaris/NM_001003318.1	TC241036	4 = 3 > 2 > 1
CF106667 (620)	Ribosomal protein L17 (*RPL17*)	B. taurus/NM_001034459.1	TC264931	4 > 3 > 2 > 1
CF106653 (407)	Ribosomal protein SA (*RPSA*)	S. scrofa/AM050292.2	TC269871	3 > 4 = 2 > 1
DW177048 (174)	Tight junction protein 1 (zona occludens 1, *TJP1*)	C. familiaris/NM_001003140.1	TC281469	2 = 3 > 1
CF106648 (228)	Tyrosine 3-monooxygenase/Tryptophan 5-monooxygenase activation protein, beta polypeptide (*YWHAB*)	H. sapiens/NM_139323.2	TC251407	3 = 4 = 2 > 1
CF106651 (310)	Wilms' tumour associating protein (*WTAP*)	H. sapiens/NM_004906.3	TC267553	4 = 3 > 2 > 1
***Nucleic Acid Metabolism Genes***				
CF106652 (492)	Basic Transcription Factor 3 (*BTF3*)	H. sapiens/NM_001207.4	TC241093	4 = 2 > 3 > 1
CF106681 (396)	DNA Polymerase Beta (*POLB*)	B. taurus/NM_001034764.1	TC252856	4 = 1 > 3 > 2
CF106666 (623)	Heterogeneous nuclear ribonucleoprotein K (*HNRPK*)	H. sapiens/NM_002140.2	TC260259	4 = 3 = 2 > 1
CF106673 (385)	La ribonucleoprotein family, member 4 (*LARP4*)	H. sapiens/NM_199190.1	TC244218	3 > 2 > 1 > 4
***Oxidative Stress Genes***				
CF106641 (364)	Flavin containing monooxygenase 3 (*FMO3*)	B. taurus/NM_174057.2	TC251612	1 > 2 = 3 = 4
CF106665 (479)	Glyoxalase I (*GLO1*)	B. taurus/NM_001083496.1	TC254694	4 > 3 > 2 > 1
CF106659 (349)	Peroxiredoxin (*PRDX4*)	B. taurus/NM_174433.2	TC239253	4 = 3 > 2 > 1
***Blood Cogulation Genes***				
CF106687 (278)	Carboxypeptidase B2 (*CPB2*)	H. sapiens/NM_001872.3	TC240093	3 = 4 > 2 = 1
CF106639 (374)	Coagulation Factor IX (*F9*)	H. sapiens/NM_000133.2	TC286008	4 > 2 > 3 = 1
CF106662 (269)	Histidine Rich Glycoprotein (*HRG*)	B. taurus/NM_173919.2	TC251128	3 > 4 > 2 = 1
***Protein and Amino Acid Metabolism Genes***				
CF106650 (196)	N-Aminoacylase I (*ACY-1*)	S. scrofa/NM_213896.1	TC269425	4 = 3 = 2 > 1
CF106684 (295)	Polyubiquitin (*UBB*)	B. taurus/NM_174133.2	TC260685	3 = 4 > 2 = 1
***Structural Genes***				
CF106657 (384)	Integrin alpha-6 (*ITGA6*)	H. sapiens/NM_000210.2	TC285515	1 > 2 = 3 = 4
***Mitochondrial DNA Genes***				
CF106643 (300)	Mitochondrial DNA	S. scrofa/AB292606.1	TC259884	1 > 3 > 2 = 4
CF106645 (177)	Mitochondrial DNA	S. scrofa/AB292606.1	TC259884	1 > 2 > 3 = 4
CF106671 (297)	Mitochondrial DNA	S. scrofa/AB292606.1	TC259884	4 = 3 > 2 > 1
CF106646 (156)	Mitochondrial DNA	S. scrofa/AB292606.1	SINGLETON	3 > 4 > 2 = 1
CF106664 (336)	Mitochondrial DNA	S. scrofa/AB292606.1	TC270954	1 > 2 > 3 = 4
CF106675 (334)	Mitochondrial DNA	S. scrofa/AB292606.1	TC270954	3 > 4 = 2 = 1
CF106647 (207)	Mitochondrial DNA	S. scrofa/AB292606.1	TC239708	3 > 4 = 2 = 1
***Unknown Identity Genes***				
CB826607 (112)^e^	No significant match		SINGLETON	1 > 4
CB826608 (288)^e^	No significant match		TC292722	1 > 4
CB826609 (250)^e^	No significant match		SINGLETON	1 > 4
CB826610 (147)^e^	No significant match		TC263361	1 > 4
CF106637 (262)	No significant match		TC269532	4 = 3 > 2 > 1
CF106638 (366)	No significant match		SINGLETON	4 = 2 > 2 > 1
CF106642 (557)	No significant match		SINGLETON	1 > 2 = 4 > 3
CF106644 (312)	No significant match		SINGLETON	1 > 2 = 3 = 4
CF106655 (211)	No significant match		SINGLETON	3 = 4 > 2 > 1
CF106656 (222)	No significant match		SINGLETON	1 = 3 > 2 > 4
CF106658 (294)	No significant match		TC288854	4 = 2 > 3 = 1
CF106660 (209)	No significant match		SINGLETON	1 > 4 = 3 > 2
CF106661 (228)	No significant match		SINGLETON	1 = 2 > 3 > 4
CF106668 (223)	No significant match		SINGLETON	1 > 2 = 3 = 4
CF106670 (225)	No significant match		TC264413	1 = 3 > 4 > 2
CF106672 (372)	No significant match		TC275435	3 > 2 > 1 > 4
CF106674 (536)	No significant match		SINGLETON	3 > 1 = 2 = 4
CF106676 (225)	No significant match		TC264413	3 = 1 > 4 > 2
CF106678 (434)	No significant match		TC284960	3 > 4 = 2 = 1
CF106682 (317)	No significant match		TC264213	3 > 4 > 2 = 1
CF106683 (364)	No significant match		TC256701	3 > 4 > 2 = 1
CF106685 (227)	No significant match		SINGLETON	1 = 4 > 2 < 3
DW177047 (247)	No significant match		TC241849	3 > 1 = 2
DW177049 (158)	No significant match		SINGLETON	2 > 1 = 3
DW177050 (133)	No significant match		TC248509	1 = 2 > 3

^a^Amplicons were compared to GenBank database using the BLASTN software v. 2.2.17 August 26, 2007.

^b^Species, GenBank accession number for most significant match.

^c^Amplicons were compared to TIGR *Sus scrofa *gene index v.12.0 updated June 20, 2006 using the BLAST software v. 2.0. Singleton denotes sequences unassigned to a TC.

^d^Pattern of transcript abundance observed on DDRT-PCR gels. 1 = Zn150; 2 = Zn1000; 3 = Zn1000P; 4 = Zn2000. Pattern observed in the display gel from highest (>) to lowest or comparable (=) abundance.

^e^Amplicons detected in Exp. 1.

### Identification of differentially expressed genes by oligonucleotide microarray analysis

To further monitor gene expression changes due to pharmacological Zn supplementation, we conducted a transcriptional profiling analysis using a commercially available pig 70-mer oligonucleotide set spotted on glass slide microarrays. The hepatic transcript abundance patterns from animals fed 150 mg Zn/kg (Zn_150_) compared to animals fed 2,000 mg Zn/kg (Zn_2000_) revealed 683 annotated genes that were significantly altered at a false discovery rate (FDR) adjusted P ≤ 0.05, plus several hundred additional un-annotated oligonucleotides. Several of these probes represent multiple spots of the same oligonucleotide. Taking this into account, a total of 650 unique genes were identified as differentially expressed at FDR ≤ 0.05 in the microarray analysis [see Additional file 1]. These genes have been categorized according to functional groups, and the file contains the oligonucleotide ID, fold change, raw and adjusted P values, and annotation for each gene.

### Confirmation of differential expression as affected by dietary treatment

In the present study, we focused on genes differentially expressed due to pharmacological Zn supplementation, and further evaluated if their expression was affected by phytase supplementation. A total of 7 genes were selected from the display gels and microarray hybridization results that exhibited different relative mRNA abundance in the liver of pigs fed pharmacological Zn diets without phytase (Zn_1000_, Zn_2000_) versus pigs fed Zn_150_. In addition to these 7 genes, an oligonucleotide for *MT *on the microarray was observed to exhibit a 3.8-fold higher hepatic mRNA abundance in pigs fed Zn_2000 _than in pigs fed Zn_150_, a result which we have already confirmed in our previous study where we reported hepatic *MT *mRNA abundance and protein concentration to be significantly higher in pigs fed pharmacological Zn diets than in pigs fed an adequate Zn diet [11]. To confirm differential gene expression, a combination of relative real time RT-PCR and northern blot analyses were utilized with all 7 animals from Exp. 1 (fed Zn_150 _or Zn_2000_without phytase), and all 23 animals of Exp.2, which were fed the six dietary treatments.

**Figure 1:**
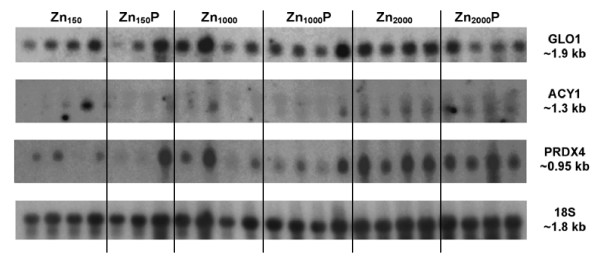
**Northern blots of *GLO1*, *ACY1 *and *PRDX4***. Northern blot analysis of liver *GLO1*, *ACY1 *and *PRDX4 *mRNA of pigs fed 150, 1,000 or 2,000 mg Zn/kg (with or without phytase) for 14 d post-weaning (Exp.2). Liver total RNA (12 μg) isolated from each pig was analyzed using the cDNA clones derived from differential display gels as probes. Also shown is *18S *rRNA hybridization to assess equivalence of RNA loading.

**Figure 2:**
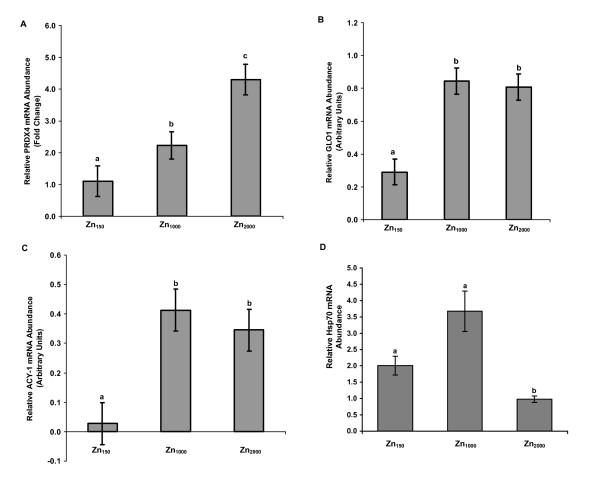
**Gene expression confirmation of DDRT-PCR and microarray transcripts**. Relative mRNA abundance of selected transcripts was confirmed using real time RT-PCR or nothern blot analyses in pigs fed 150, 1,000 or 2,000 mg Zn/kg for 14 d post-weaning. **A) *PRDX4 *mRNA Abundance**. Relative real time RT-PCR was performed and fold changes relative to *GAPDH *and a common Zn_150 _reference sample are presented. Values are means ± SEM, n = 8, (except Zn_150_, n = 7). A significant zinc effect was detected for relative *PRDX4 *mRNA abundance, *P *< 0.009. No Zn by phytase interaction, or phytase effect were detected. **B) *GLO1 *mRNA Abundance**. Northern blot analysis was performed and values are mean optical density readings ± SEM, n = 8 (except Zn_150_, n = 7). A significant zinc effect was detected for relative hepatic *GLO1 *mRNA abundance, *P *< 0.0007. No Zn by phytase interaction, or phytase effect were detected. **C) *ACY1 *mRNA Abundance**. Northern blot analysis was performed and values are mean optical density readings ± SEM, n = 8 (except Zn_150_, n = 7). A significant zinc effect was detected for relative hepatic *ACY1 *mRNA abundance, *P *< 0.01. No Zn by phytase interaction, or phytase effect were detected. **D) *HSP70.2 *mRNA Abundance**. Relative real time RT-PCR was performed and fold changes relative to Zn_2000 _using *18S *as normalizing gene are presented. Values are means ± SEM, n = 7, (except Zn_2000_, n = 8). A significant zinc effect was detected for relative *HSP70.2 *mRNA abundance, *P *< 0.002. No Zn by phytase interaction, or phytase effect were detected.

Three oxidative stress response genes involved in the reduction of peroxides [*peroxiredoxin 4 *(*PRDX4*)], detoxification of glycating agents [*glyoxalase I *(*GLO1*)], and transfer of glutathione [*glutathione transferase M4 (GSTM4)*], exhibited differential expression. Northern blot analysis of *PRDX4 *revealed a single transcript of ~0.95 kb (Figure 1), which agrees with the size reported for human *PRDX4 *[20]. Real time reverse transcription PCR (RT-PCR) was used to examine mRNA abundance of *PRDX4*. Relative abundance of *PRDX4 *mRNA (Figure 2A) in liver of pigs fed pharmacological Zn diets was 2 fold higher in pigs fed Zn_1000_, and 4 fold higher in pigs fed Zn_2000 _when compared to pigs fed Zn_150 _(*P *< 0.009). This result was supported by the subsequent microarray analysis which revealed significantly higher hybridization to a PRDX4 oligonucleotide present on the array by the Zn_2000 _samples than by the Zn_150 _samples (data not shown).

PCR amplification efficiency plots revealed that *GAPDH *was not a suitable normalizing gene for *GLO1*, therefore northern blot hybridization was performed for confirmation of *GLO1*. A single transcript (~1.9 kb) was observed (Figure 1), which was in accordance with the size of human *GLO1 *[21]. Relative *GLO1 *mRNA abundance was greater (*P *< 0.0007) in pigs fed pharmacological Zn diets (Zn_1000 _and Zn_2000_) when compared to pigs fed adequate Zn (Figure 2B). Supplemental phytase did not affect the mRNA abundance of either *PRDX4 *or *GLO1*.

Microarray analysis had indicated *GSTM4 *mRNA abundance to be higher in pigs fed Zn_2000 _than pigs fed Zn_150_. Real time RT-PCR results for *GSTM4 *supported a trend for Zn_2000 _pigs to have higher *GSTM4 *mRNA abundance, although it did not reach statistical significance due to large animal variability. There was a trend for significance (*P *< 0.09) towards an interaction between Zn and phytase supplementation for relative *GSTM4 *mRNA abundance. *GSTM4 *mRNA abundance tended to increase when phytase was supplemented to the diets of pigs fed adequate (Zn_150_P) or the first level of pharmacological Zn (Zn_1000_P). However, when phytase was supplemented with the higher level of pharmacological Zn (Zn_2000_P) no further increase in expression was observed.

*N-aminoacylase I *(*ACY1*) and *heat shock protein 70.2 (HSP70.2) *are genes involved in amino acid and heat shock metabolism that also displayed differential expression. Northern blot analysis revealed the presence of a single *ACY1 *transcript (~1.3 kb, Figure 1), which is in accordance with the size reported for pig *ACY1 *[22]. Relative *ACY1 *mRNA abundance was increased (*P *< 0.01) in pigs fed pharmacological Zn (Zn_1000 _and Zn_2000_), and no phytase effect was observed (Figure 2C). Relative abundance of *HSP70.2 *mRNA in liver of pigs fed Zn_150 _and Zn_1000 _diets was comparable and higher than in pigs fed Zn_2000 _(Figure 2D). Supplemental phytase did not affect the mRNA abundance of *HSP70.2*.

Based on the differential display gels, an acute phase response gene, *orosomucoid 1 *(*ORM1*), and a gene in the plasma carboxypeptidase family, *carboxypeptidase B2 *(*CPB2*), also exhibited increased mRNA abundance in pigs fed pharmacological Zn diets. However, these genes were not confirmed to be differentially expressed in pigs fed any of the dietary treatments using relative real time RT-PCR due to large animal variability (data not shown).

## Discussion

Differential display reverse transcription PCR is a technique that allows the simultaneous comparison of multiple treatments for the identification of differentially regulated mRNAs, and the generation of ESTs that can be compared with DNA sequences available in current databases to identify genes, including genes in non-conventional biological models such as the pig [23,24]. The disadvantage of DDRT-PCR vs. microarray approaches is clearly that it is a much more time consuming technique to perform. However, DDRT-PCR does have some advantages. Gene discovery with DDRT-PCR does not require prior knowledge of gene or EST sequences as is needed for construction of microarrays [25]. In the present study, several genes selected for confirmation from the DDRT-PCR study were not present on the microarray used for subsequent analyses despite the relatively large number of oligonucleotides on the microarray (over 13,000). In addition, DDRT-PCR allows direct comparisons to be made between more than two samples at a time and it may be more sensitive for detection of relatively low abundance transcripts. For this study, DDRT-PCR and oligonucleotide microarray technologies were used to reveal differentially expressed genes in the liver of pigs fed pharmacological Zn diets and to determine if expression was further affected when supplemental phytase was added to the diets. In total we found 61 ESTs with putative differential expression using DDRT-PCR among the various treatment groups and identified 650 differentially expressed (FDR ≤ 0.05) annotated genes with the microarray. Seven genes were selected for confirmation. This study provides evidence of a differential hepatic transcriptional profile in pigs due to consumption of diets containing basal or pharmacological concentrations of Zn.

The *PRDX4 *gene encodes for a ubiquitous cytosolic enzyme [EC 1.11.1.15], which reduces hydrogen peroxide via redox-active cysteine residues. In addition to hydrogen peroxides, Jin and collaborators [20] reported that peroxiredoxins can also regulate peroxide-mediated signaling cascades, and their overexpression reduces hydrogen peroxides in response to tumor necrosis factor α (TNF-α). Overexpression of PRDX4 in mice suppresses B-cell nuclear factor of kappa light polypeptide gene enhancer (Nf-κB) activation through regulation of B-cell inhibitor of kappa light polypeptide gene enhancer (I-κB) phosphorylation [20]. Gallagher and Phelan reported that inhibition of Nf-κB leads to an increase in *PRDX6 *transcription in mouse hepatocytes [26]. Similarly, MT has also been implicated in inhibiting TNF-induced activation of Nf-κB DNA binding, by inhibition of I-κB degradation and subsequent NF-κB suppression [27]. An *in vitro *study demonstrated that Zn pretreatment increased the availability of intracellular Zn ions and abrogated LPS-induced oxidative stress and I-κB/NF-κB signaling cascade in liver Kupffer cells [28]. Kim et al. [29] suggest that MT modulates intracellular signaling molecules such as transcription factors (Nf-κB) by sequestering intracellular Zn, which is required for its DNA binding activity. Peroxiredoxins are considered redox regulators of signal transduction [20], acting like a switch to control intracellular pathways. Perhaps the increase in intracellular Zn causes an alteration in cell signaling [30], and PRDX4 upregulation occurs as a result. In addition, the suppressive effect of PRDX4 on Nf-κB may be one of the many mechanisms of how pharmacological Zn, as ZnO enhances health in the newly weaned pig. For example, studies in Caco-2 cells exposed to enterotoxigenic *E. coli *demonstrate that Zn as ZnO protects these cells by inhibiting bacterial adhesion and internalization into cells, thus modulating subsequent cytokine expression that might occur in response to pathogenic exposure [31].

Glyoxalase 1 [EC 4.4.1.5] is a cytosolic ubiquitous Zn metalloenzyme that catalyzes the glutathione dependent conversion of glycating agents such as methylglyoxal to S-D-lactoylglutathione via a 1,2-hydrogen transfer [32]. This enzyme works in conjunction with glyoxalase II to further convert S-D-lactoylglutathione to D-lactate [33]. Methylglyoxal can react with arginine and lysine residues in proteins, and at high concentrations it inhibits glycolytic enzymes [21], enhances lipid peroxidation, and binds DNA and RNA causing macromolecular damage [34]. Genomic analysis of the *GLO1 *gene revealed the presence of functional metal response elements (MREs) located 647 bp downstream of the transcription initiation site [21]. Another gene containing MREs in its promoter region is the *MT *gene [35]. The MREs control transcriptional activation of *MT *upon exposure to Zn, through the action of metal transcription factor-1 [36]. Transcriptional activity of *GLO1 *in transfected HepG2 cells was elevated twofold upon exposure to 25 and 75 μM ZnCl_2_for 48 hr [21]. We have previously shown that *MT *mRNA abundance and protein concentration are increased in liver, kidney and intestinal mucosa of pigs fed pharmacological Zn [11]. This observation was confirmed for liver *MT *mRNA abundance with the microarray analysis in the present study and similar expression patterns were observed for *PRDX4 *and *GLO1*.

Zinc involvement in oxidative stress is well known. Its deficiency causes oxidative DNA damage by impairment of antioxidant defense, and compromises DNA repair mechanisms [37]. However, the mechanism of how supplemental Zn exerts its antioxidant action is not well defined. Our current microarray analysis indicated that mRNA abundances of many oxidative stress molecules are affected by pharmacological Zn supplementation including *GSTM4*, glutathione peroxidase (*GPX*), NADPH oxidase (*NOX1*), heme oxygenase 2 (*HMOX2*), cytochrome C oxidase VIIB (*COX7B*), cytochrome P450 (*CYPC27*), and peroxisome proliferator activated receptor gamma-2 (*PPARG*) [see Additional file 1]. From these, *GSTM4 *was selected for confirmation. *GSTM4 *encodes the fourth member of the glutathione S-transferase that belongs to the mu class. The mu class of enzymes function in the detoxification of electrophilic compounds, including carcinogens, therapeutic drugs, environmental toxins and products of oxidative stress, by conjugation with glutathione [38]. Our results are in agreement with previous studies where dietary Zn affected the hepatic gene expression of *GST, GPX *and *CYP *[39]. In addition to these genes, it has been suggested that *MT *induction by Zn in hepatocytes creates an intracellular pool of thiols and Zn ions, thus it can act similarly to glutathione which can then react with the reactive species [40,41]. Moreover, the ability of Zn to occupy iron and copper binding sites on lipids, proteins and DNA, may also contribute towards its antioxidant action [42]. The present study shows that pharmacological Zn supplementation is associated with increased mRNA abundance of *PRDX4 *and *GLO1*, and a tendency to increase *GSTM4 *when combined with dietary phytase. Interestingly these genes participate in the oxidative stress response through differing mechanisms. Perhaps the increased intracellular concentrations of Zn exert an increase in Zn dependent enzymes creating an effective hepatoprotective role. Our results for hepatic *GLO1 *expression are in agreement with observations in *GLO1 *transfected HepG2 cells [21]. It is possible that Zn transcriptionally regulates *GLO1 *through MRE activation, in a similar fashion to the *MT *gene, therefore offering protection against glycating agents. However, to our knowledge, we provide the first evidence that dietary pharmacological Zn increases *PRDX4 *and *GLO1 *mRNA abundances. Additional studies will be undertaken to further clarify the influence of pharmacological Zn on *GSTM4 *and related oxidative stress protein activity in pigs.

Molecular chaperones or stress proteins like heat shock proteins 70.2 and 90 (Hsp70 and Hsp90), maintain the correct conformational homeostasis of proteins, helping newly synthesized proteins to be folded and transported across membranes and protecting them against a number of stresses thus playing the role of chaperones [43,44]. The expression of heat shock proteins is under complex control, but with final common pathways involving the heat shock factors (HSFs), the function of which is zinc-dependent [45]. Several heat shock proteins have been identified including Hsp27, Hsp47, Hsp70 and Hsp90, and are named according to their molecular weight [46]. In our present study we were able to confirm that pharmacological Zn decreases *HSP70.2 *mRNA abundance in the liver. Interestingly, *in vitro *studies [47] which examined the relationship between protein misfolding, aggregation and chaperone induction showed that oxidative stress increased expression of Hsp90. However, mild oxidative stress did not induce protein denaturation and stress response. This work also showed that adding Zn induced chaperone expression and higher Zn concentrations counteracted the heat shock and oxidative stress mediated induction of chaperones by inhibiting their expression [47]. These studies support our findings that at higher Zn concentrations, there is lower abundance of *HSP70.2 *mRNA.

Three additional genes were selected for confirmation. *ACY1 *encodes for a Zn metalloenzyme involved in peptide metabolism, *ORM1 *encodes for an acute phase reactant protein and *CPB2 *encodes for a plasma carboxypeptidase. ACY1 [EC 3.5.1.14] is a cytosolic enzyme with highest abundance in pig kidney versus the liver [22,48]. The biological role of ACY1 is to hydrolyze neutral and hydrophobic α-*N*-acyl-L-amino acids generated during protein degradation [49]. This Zn dependent enzyme has been identified in mammals primarily in kidney and liver [48], it contains a single Zn atom per subunit, and it is proposed to have catalytic roles [50]. However, Heese et al. [51] suggested that Zn plays a structural role, because the Zn binding site was too far from the catalytic site of the enzyme. Results of northern blot analysis in this study confirm that pharmacological Zn supplementation increases the relative abundance of *ACY1 *mRNA. No differences were observed in the abundance of this transcript between animals fed Zn_1000 _and Zn_2000_, suggesting that at dietary concentrations greater than 1,000 mg Zn/kg, relative *ACY1 *abundance reaches a plateau. To our knowledge, this study provides the first evidence of Zn effects on the abundance of *ACY1 *mRNA, suggesting a role in protein and amino acid metabolism.

*Orosomucoid 1 *and *CPB2 *are genes that encode an acute phase reactant protein known as α-1-acid glycoprotein, and plasma pro-carboxypeptidase [EC 3.4.17.20], respectively. These plasma proteins are secreted into the blood after being synthesized in the liver [52,53]. Serum concentrations of ORM1 are high in fetal [54] and neonatal pigs, decreasing markedly to a constant level by 2 wk of age [55] and declining further by 112 d. In a similar pattern, liver *ORM1 *mRNA is relatively abundant in late fetal and neonatal pigs then declines rapidly after birth [56]. Alpha-1-acid glycoprotein plasma concentration rises during inflammation, thus its function is related to modulation of an inflammatory response primarily caused by interleukin 1, interleukin 6 and glucocorticoids [57]. Sequence analysis of the murine *ORM1 *gene revealed the presence of four MRE sequences in the 5' flanking region, and one in intron 5 [58]. Furthermore, relative liver *ORM1 *mRNA abundance in mice injected i.p. with the heavy metal mercury (0.5 mg Hg/kg) was increased in a time dependent manner. Zinc was also used to investigate *ORM1 *mRNA response upon heavy metal exposure, but its effect on *ORM1 *mRNA was not as marked as Hg [59]. The authors concluded that heavy metals regulate *ORM1 *at the transcriptional level. Interestingly, we found this gene to be affected by dietary treatment in both experiments using different anchor and arbitrary primer combinations, although the pattern of transcript abundance differed. We did not confirm a significant effect of dietary Zn on liver *ORM1 *mRNA abundance for pigs in either experiment using relative real time RT-PCR.

Plasma CPB2 [E.C. 3.4.17.20] is a carboxypeptidase that appears in plasma after clotting of blood [60]. Several names have been used for this enzyme according to its general catalytic mechanism, including carboxypeptidase U (U = unstable), carboxypeptidase R (R = cleaves Arg and Lys residues in C-termini), plasma carboxypeptidase B (B = basic) and most recently thrombin-activatable fibrinolysis inhibitor (TAFI) [61]. Eaton et al. [62] showed that CPB2 becomes inactive upon addition of a Zn chelating agent, 1,10-phenantroline. The presence of CPB2 has been demonstrated in the pig, rabbit and mouse, with activity ranging from 20% (mouse) to 500% (pig) compared to human serum CPB2 [63]. The physiological function of CPB2 is to inhibit fibrinolysis by removing C-terminal lysine residues from fibrin and limiting plasmin formation [64]. Fibrinogen is also secreted during an inflammatory response, suggesting that overexpression of CPB2 could be part of a mechanism to decrease inflammation.

Our display gels indicated that pharmacological Zn increases the mRNA abundance of *ORM1 *(for Exp. 2 pigs) and *CPB2 *in pig liver. However, relative real time PCR results did not confirm their differential expression. The inability to confirm the differential display observations may be due to the low number of pigs examined along with relatively high variability in transcript abundance between pigs or the increased sensitivity of the real time RT-PCR assays.

## Conclusion

The EST data obtained and submitted to the GenBank database as well as the transcriptional profiling from the microarray study provide sequence and differential gene expression information for hepatic genes in the pig. In addition, this study provides important information that contributes to understanding the benefits of feeding pharmacological Zn diets to pigs. New mechanisms affected by dietary Zn were evaluated and can be further considered in future studies, including the role of Zn in reducing oxidative stress and protein metabolism, both processes that are essential for cell detoxification and function.

## Methods

### Animals and diets

Animals and diet composition have been previously described [11]. A smaller preliminary study was performed to optimize the technique (Exp. 1). For Exp.1 the dietary treatments fed to pigs for 14 d were: 1) basal Zn diet containing 150 mg Zn/kg (Zn_150_), 2) pharmacological Zn diet containing 2,000 mg Zn/kg (Zn_2000_). The dietary treatments fed to pigs in Exp. 2 for 14 d were: 1) adequate Zn diet containing 150 mg Zn/kg (Zn_150_), 2) Zn_150 _plus 500 phytase units (FTU)/kg (Zn_150_P), 3) pharmacological Zn diet containing 1,000 mg Zn/kg (Zn_1000_), 4) Zn_1000 _plus 500 FTU/kg (Zn_1000_P), 5) pharmacological Zn diet containing 2,000 mg Zn/kg (Zn_2000_), or 6) Zn_2000 _plus 500 FTU/kg (Zn_2000_P). Pigs were provided feed and water *ad libitum*. This project was approved by the Michigan State University All University Committee on Animal Use and Care (12/99-159-00).

### Sample collection and total RNA isolation

Details about euthanasia, liver sample collection and total RNA isolation were previously published [11]. Liver samples were obtained from 3 pigs per treatment for Exp. 1 (n = 6 total) and from 4 pigs per treatment for Exp. 2 (n = 24 total). RNA quality and integrity were determined by calculating the A_260/280 _ratio and by agarose gel electrophoresis, respectively. Additionally, RNA quality and quantity were further confirmed with the RNA 6000 Pico LabChip^® ^kit using an Agilent 2100 Bioanalyzer (Agilent Technologies, Palo Alto, CA).

### Differential display reverse transcription polymerase chain reaction (DDRT-PCR)

DDRT-PCR experiments were performed as previously reported by our laboratory [65] using modifications of published procedures [23]. Briefly, genomic DNA contamination was minimized by treating 1 μg of total RNA with 1 U of amplification grade DNAse I (Invitrogen, Life Technologies, Carlsbad, CA) according to the manufacturer's protocol. Individual DNAse treated RNA samples (200 ng) from animals fed Zn_150 _and Zn_2000 _(n = 3 per treatment in Exp.1) and fed Zn_150_, Zn_1000_, Zn_1000_P and Zn_2000_, (n = 4 per treatment in Exp. 2), were reverse transcribed by using anchor primers (Exp. 1 – Anchors #2 and #7, Exp.2 – Anchor #9) and RT-mix [1× Buffer, 25 μM dNTPs, 10 mM DTT and 40 U of Superscript II (Invitrogen, Life Technologies, Carlsbad, CA)]. Samples were incubated at 40°C for 5 min, 50°C for 50 min, and 70°C for 15 min in a Peltier Thermal Cycler (PTC-200, MJ Research). A final temperature drop to 4°C stopped the reaction.

Oligonucleotide primers for DDRT-PCR were obtained from the U.S. Pig Genome Coordination Program , and those used for these experiments were randomly selected (Table 2). For Exp. 1, 2 anchor primers were paired with 2 arbitrary primers on 6 cDNA samples and a total of 24 PCR amplicons were generated. For Exp. 2, 1 anchor primer paired with 6 arbitrary primers on 16 cDNA samples resulted in a total of 96 PCR amplicons, thus ~5% of all mRNA species present were screened. PCR reactions were performed using cDNA (400 ng) in a solution containing 0.2 μM 3'-anchor primer, 20 μM of each dNTP, 1.5 mM MgCl_2_, 1× PCR buffer, 0.2 μM 5' – arbitrary primer, 2.5 μCi [α-^33^P] dATP (Perkin Elmer, Life Sciences Inc., Boston, MA) and 0.5 U Taq DNA Polymerase (Promega, Madison, WI). PCR cycling parameters were: 95°C for 2 min, four cycles at 92°C for 15 s, 50°C for 30 s, and 72°C for 2 min, followed by an additional 25 cycles with annealing at 60°C for 30 s and extension at 72°C for 2 min.

**Table 2:** Anchor and arbitrary primers^a^.

**Primer identification number**	**Primer sequence**
Anchor # 2 d(T)_12 _AC^b^	5'-TTTTTTTTTTTTGC-3'
Anchor # 7 d(T)_12 _AC^b^	5'-TTTTTTTTTTTTCG-3'
Anchor # 9 d(T)_12 _AC^c^	5'-TTTTTTTTTTTTAC-3'
Arbitrary # 4^c^	5'-GCTAGCAGAC-3'
Arbitrary # 7^c^	5'-TGGATTGGTC-3'
Arbitrary # 9^c^	5'-TAAGCCTAGC-3'
Arbitrary # 13^c^	5'-GTTGCACCAT-3'
Arbitrary # 14^b^	5'-TCCATGACTC-3'
Arbitrary # 16^d^	5'-TCGGTCATAG-3'
Arbitrary # 18^b^	5'-TGATGCTACC-3'
Arbitrary # 20^c^	5'-TCGATACAGG-3'

^a^Source: .

^b^Used in Exp. 1.

^c^Used in Exp. 2.

^d^Used in Exp. 1 & 2.

Denaturing loading dye (96% formamide, 2% bromophenol blue and 2% xylene cyanol) was mixed with each DDRT-PCR sample (8 μl), and samples were dried on medium heat for 5 min using a speed-vacuum (Savant Instruments Inc., Farmingdale, NY) followed by a 3 min 95°C denaturing step. The samples were electrophoresed on 0.4 mm 5.2% polyacrylamide denaturing gels. Following a 5–6 hr run at 60 W on a vertical gel box (Model S2, Invitrogen, Life Technologies/GIBCO BRL Sequence Systems, Carlsbad, CA), gels were transferred onto Whatman chromatography paper (Whatman^®^, Maidstone, England) and dried at 80°C for 30 min using a slab dryer (SGD 2000, Savant). Gels were exposed to Biomax™ film (Eastman Kodak Co., Rochester, NY) overnight.

### Excision and re-amplification of DDRT-PCR products

Radiographs were developed and subsequently carefully evaluated to detect differences in transcript abundance by visually comparing bands among the different dietary treatments. Transcripts were selected that exhibited a consistent band intensity among samples within treatments and different intensities across treatments. A band intensity scale to describe the pattern of transcript abundance was created by assigning a representative number to each dietary treatment (1 = Zn_150_; 2 = Zn_1000_; 3 = Zn_1000_P; 4 = Zn_2000_) observed in the display gel from highest (>) to lowest or comparable (=) abundance (Table 1). Selected bands were circumscribed from the gels, re-hydrated in 100 μl DEPC-treated water and heated at 50°C for 30 min. Re-amplification reactions included 2 μl of gel band eluate and the anchor and arbitrary primers used in the DDRT-PCR step under the same reaction conditions and cycling parameters described above, excluding the isotope. To assess quality of the reactions, 1 and 2 μl of re-amplified products were electrophoresed with a λ *Hind *III marker (Invitrogen, Life Technologies, Carlsbad, CA) in 1% agarose gels stained with ethidium bromide (Sigma-Aldrich, St. Louis, MO).

### Cloning and sequencing of DDRT-PCR products

Products of interest obtained from the DDRT-PCR gels were cloned into pGEM-T-Easy Vector-System I (Promega, Madison, WI). Recombinant vectors were transformed into *E. coli *DH5α competent cells (Invitrogen, Life Technologies, Carlsbad, CA). Plasmid DNA was purified with the QIAprep Spin Miniprep kit (Qiagen, Valencia, Ca) and digested with *EcoR *I to confirm the presence of an insert. Sequencing was performed using SP6 or M13 forward primers at the Michigan State University Research Technology Support Facility (MSU RTSF). Putative identification of the amplicons was determined using the basic local alignment search tool (BLASTn) software, with the non-redundant and EST databases of GenBank and The Institute for Genomic Research (TIGR) database. Correct gene name and abbreviation was corroborated using the Gene Cards^® ^database .

### Oligonucleotide microarray analysis

Oligonucleotide microarrays used for this study consisted of 13,297 70-mer oligos (Pig Array-Ready Oligo Set v. 1.0 and Pig Oligo Extension Set v. 1.0, Qiagen, Inc., Valencia, CA) each spotted once on a single slide. Slides were printed at the MSU RTSF. Oligonucleotides spotted in multiple locations for use as potential controls included 76 Arabidopsis thaliana gene spots, 17 beta tubulin spots, 17 glyceraldehyde-3-phosphate dehydrogenase spots, 85 heat shock protein gene spots, 69 ribosomal protein gene spots, 112 randomly generated negative control spots and 470 blanks. This microarray has been validated for use in pig transcriptional profiling studies by Zhao et al. [66]. The microarray was screened with the Zn_150 _and Zn_2000 _samples. Zn_150 _samples were randomly paired with Zn_2000 _samples using four microarray slides. Two samples from each treatment were labeled with Cy3 and the other two were labeled with Cy5.

For each sample, 8 μg of total RNA was reverse transcribed with an oligo (dT)_18 _primer using the Superscript™ Indirect cDNA Labeling System (Invitrogen, Carlsbad, CA) according to the manufacturer's instructions. After first-strand synthesis and purification, the cDNAs incorporated amino-modified dUTPs and were labeled with *N*-hydroxysuccinate (NHS) ester Cy3 or Cy5 dyes (GE Healthcare, Piscataway, NJ). The labeled cDNAs were purified, combined and concentrated to 10 μl using a microcon spin column (Millipore, Bedford, MA). The concentrated probe was combined with 100 μl of Slide Hyb#3 solution (Ambion, Inc. Austin, TX) and denatured at 70°C for 5 min. Microarray hybridizations took place in sealed hybridization chambers in a GeneTAC™ Hybridization Station (Genomic Solutions) for 18 hours using step-down temperatures ranging from 65°C to 42°C. Following hybridization, the slides were washed twice with medium stringency buffer and once with high stringency buffer (Genomic Solutions). The slides were rinsed in 2 × SSC and deionized water and were dried using centrifugation at 1000 × g for 2 min. Fluorescent images were detected on a GenePix 4000B scanner (Molecular Devices, Sunnyvale, CA). Fluorescence intensity data were collected using GenePix software (Molecular Devices) after spot alignment. The dataset was submitted to the National Center for Biotechnology Information's Gene Expression Omnibus database [GEO:GSE11972]. Total intensity values for each dye channel were stored as comma-separated values data files and exported into Microsoft Excel spreadsheets for subsequent analysis. The fluorescence intensity data was background corrected and analyzed using the limma software [67]. Specifically, the "normexp" function advocated by Smyth [67] was used for background subtraction followed by print-tip specific loess normalization for dye intensity bias for each array. The Cy5:Cy5 log2 ratios were further scale normalized between arrays. A simple linear model fitting these ratios as a function of an overall intercept(dye) and treatment effects was fitted for each gene using the "eBayes" option in limma. Statistical significance was based on the estimated false discovery rates (FDR) as also provided by limma.

### Independent confirmation by relative real time RT-PCR

Transcript abundance of *ORM1 *was examined for liver samples from Exp.1 (n = 4 fed Zn_150 _and n = 3 fed Zn_2000_). Transcript abundances of 7 genes (*ACY1*, *GLO1*, *PRDX4*, *CPB2*, *ORM1, GSTM4 *and *HSP70.2*) were evaluated for 23 liver samples from Exp. 2. The RNA sample for 1 pig fed Zn_150_P (Exp.2) was of poor quality, and was not included in the analyses. *Glyceraldehyde-3-phosphate dehydrogenase *(*GAPDH*) or *18S ribosomal RNA *(*18S*) were used as normalizing genes. Relative real time RT-PCR primers (Table 3) were designed using Primer Express v. 2.0 (Applied Biosystems, Foster City, CA), and the assays were performed on an ABI Prism 7000 Sequence Detection System (Applied Biosystems, Foster City, CA) in the Michigan State University Center for Animal Functional Genomics. First strand cDNA synthesis was performed with an oligo (dT)_14 _primer using SuperScript II RNase H (Invitrogen Life Technologies) following the manufacturer's protocol. The cDNA was purified with QuickClean resin (Clontech) followed by precipitation with sodium acetate and ethanol. Purified cDNAs were suspended in DNase/RNase-free sterile water, and quantified using a spectrophotometer. The cDNA samples were diluted to a final concentration of 10 ng/μl and stored at -20°C until use. The real time RT-PCR reactions included 50 ng of cDNA, 300 μM primer and 1× SYBR Green Master Mix (Applied Biosystems, Foster City, CA).

**Table 3:** Real time RT-PCR primers used for DDRT-PCR and microarray confirmation^a^.

Gene	Direction	Primer sequence
CPB2	Forward	5'-TGG CAT GTC ATC AGA AAT GGT T-3'
CPB2	Reverse	5'-CTT GCT GGA ATC AGT AAA TTT CAC TCT-3'
GSTM4	Forward	5'-CCA TCC TGC GCT ACA TTG C-3'
GSTM4	Reverse	5'-CTC CAA AAC ATC CAC TCG AAT CT-3'
HSP70.2	Forward	5'-GTT CGG TTT CCG GCT TCA-3'
HSP70.2	Reverse	5'-CTC TCT CCG CAA ACA GCC TCT A-3'
ORM1	Forward	5'-TTG AGT GCA CGG GAA TCC A-3'
ORM1	Reverse	5'-CCA GCG GCC CAC ACA-3'
PRDX4	Forward	5'-ATG ACC TCC CTG TGG GTA GAT CT-3'
PRDX4	Reverse	5'-ACA GAC TTC TCC ATG TTT GTC AGT GT-3'
GAPDH	Forward	5'-TGG AAA GGC CAT CAC CAT CT-3'
GAPDH	Reverse	5'-CCA GCA TCG CCC CAT TT-3'
18S^b^	Forward	5'-CGG CTA CCA CAT CCA AGG AA-3'
18S	Reverse	5'-GCT GGA ATT ACC GCG GCT-3'

^a^Primers were designed using Primer Express v. 2.0 (1995–2000).

^b^18S primers were graciously provided by Dr. Susan Ewart of the Molecular Respiratory and Equine Genetics Laboratory at Michigan State University.

Relative quantification was determined using duplicate cDNA samples from each animal. PCR amplification efficiency plots were generated using serially diluted cDNA (4 dilutions) to confirm the use of *GAPDH *or *18S *as normalizing genes for each primer pair. Results were recorded relative to a common liver cDNA sample from a pig fed Zn_150 _after normalizing for *GAPDH *or *18S*. Relative gene expression changes were then computed using the 2^-ΔΔCt ^method [68].

### Independent confirmation by northern blot hybridization

When PCR amplification plots did not reveal equal amplification efficiency for *GAPDH *with the target gene, confirmation of DDRT-PCR results for some genes was done by northern blot analysis using the cDNA clones obtained by DDRT-PCR, labeled as described previously [11] with an *18S *rRNA for normalization.

### Statistical analysis

The data were analyzed using analysis of variance based on the MIXED procedure of SAS [69]. If the main effects (Zn or phytase) or their interaction were not statistically significant, the datasets were merged. For the relative real time RT-PCR analyses, the model included pig nested within the treatment interaction with the plate as random effect. Satterthwaite's approximation was used to determine the error df for test [70]. A base 2 logarithmic transformation (Log_2_) of the target and normalization gene Cts was made to account for the exponential amplification of cDNA prior to analyzing Ct values with the MIXED procedure of SAS. The transformed target Cts, and *GAPDH *or *18S *Cts modeled as covariates, were used in a regression-based normalization of Cts in accordance with recommendations by Poehlman [71]. These values were compared to the results calculated by using the 2^-ΔΔCt ^method [68], and data is presented as fold changes. For northern blot analyses, the blot by treatment interaction was used in the model with pig as a random effect. The *18S *rRNA values were modeled as covariates for a regression-based normalization of the genes' relative mRNA abundance. Differences were considered to be significant when *P *< 0.05.

## Authors' contributions

MMM performed the animal study, prepared and analyzed the diets, helped in tissue collection, carried out part of experiment 2 DDRT-PCR reactions, performed most of the bioinformatics and statistical analyses, performed part of the confirmation assays and prepared the manuscript. GMH obtained the animals for this study, helped in obtaining the tissues, designed and coordinated the nutrition study as the PI, and helped draft the manuscript. NER helped in tissue collection, performed experiment 1 DDRT-PCR analysis, conducted part of the real time RT-PCR confirmation, and helped draft the manuscript. VDR performed part of the real time RT-PCR confirmation and conducted some of the real time RT-PCR statistical analyses. RJT performed statistical analysis of the microarray data and assisted with analysis of real time RT-PCR data. JEL prepared and analyzed the nutritional composition of the diets and helped with tissue collection. CJW carried out part of experiment 2 DDRT-PCR reactions. AMR carried out the microarray hybridizations. CWE helped in obtaining the tissues, designed and coordinated the DDRT-PCR and microarray studies as the PI, and prepared the manuscript. All authors have read and approved the final manuscript.

## Supplementary Material

Additional file 1 **Pharmacological zinc supplementation-related changes in mRNA abundance in pig liver determined by oligonucleotide microarray analysis (FDR < 0.054).** The data provided are results of oligonucleotide microarray analysis comparing pigs fed an adequate Zn diet (150 mg Zn/kg) to pigs fed a pharmacological Zn diet (2,000 mg Zn/kg). Click here for file
